# Antibiotic resistance alters through iron-regulating Sigma factors during the interaction of *Staphylococcus aureus* and *Pseudomonas aeruginosa*

**DOI:** 10.1038/s41598-021-98017-5

**Published:** 2021-09-16

**Authors:** Hamed Tahmasebi, Sanaz Dehbashi, Mohammad Reza Arabestani

**Affiliations:** 1 School of Medicine, Shahroud University of Medical Sciences, Shahroud, Iran; 2 Department of Microbiology, Faculty of Medicine, Hamadan University of Medical Sciences, Hamadan, Iran

**Keywords:** Microbiology, Medical research

## Abstract

Iron is a limiting factor in such a condition that usually is sequestered by the host during polymicrobial infections of *Pseudomonas aeruginosa* and *Staphylococcus aureus*. This study aimed to investigate the interaction of *S. aureus* and *P. aeruginosa*, which alters iron-related sigma factors regulation and antibiotic resistance. The antibiotic resistance of *P. aeruginosa* and *S. aureus* was investigated in a L929 cell culture model. The expression level of *pvdS*, *hasI* (*P. aeruginosa* sigma factors), and *sigS* (*S. aureus* sigma factor) genes was determined using Quantitative Real-Time PCR. *pvdS* and *hasI* were downregulated during co-culture with *S. aureus*, while the susceptibility to carbapenems increased (p-value < 0.0001). Also, there was a direct significant relationship between resistance to vancomycin with *sigS*. Regarding the findings of the current study, iron-related sigma factors of *P. aeruginosa* and *S. aureus* play a role in induction susceptibility to various antibiotics, including carbapenems and vancomycin.

## Introduction

As the skin integrity is lost, the subcutaneous skin layer provides a suitable condition (moisture, temperature, and nutrients) for colonization, wound infection, and biofilm formation^1^.

Microorganisms involved in polymicrobial infections compete for colonization, nutrients (including iron, manganese, copper, and zinc), and pathogenicity. As a micronutrient, iron plays a critical role in biofilm formation, Quorum sensing (QS), extracellular matrix (ECM) production, and antibiotic susceptibility^2,3^. Therefore, microorganisms develop different mechanisms, such as siderophores and heme assimilation factors, to acquire iron from the environment. *Staphylococcus aureus* and *Pseudomonas aeruginosa,* as dominant microorganisms involved in the wounds' polymicrobial infections, develop different strategies to acquire iron, including pyoverdine and pyochelin in *P. aeruginosa* and Isd proteins in *S. aureus*. During chronic infection, the dependence on siderophores decreases. In such a situation, due to iron limitation, *S. aureus* and *P. aeruginosa* shift to heme and hemoglobin to provide iron through hemophores^4,5^. As a negative regulator, Ferric Uptake Regulator (Fur) controls the expression of proteins required for iron uptake and transport in both *P. aeruginosa* and *S. aureus*. Moreover, the iron acquisition is regulated by extracytoplasmic function sigma factors (ECF), including *hasI* and *pvdS* in *P. aeruginosa*. However, less is known about *S. aureus* sigma factors' relationship to iron acquisition. ECF sigma factors regulating iron metabolism may play a role in antibiotic resistance during coinfections^6,7^.

Also, the interaction of *S. aureus* and *P. aeruginosa* may result in alteration of the strains` phenotype, persistence, and selection of Small Colony Variants (SCV). Persisters and SCVs are tolerant against aminoglycosides and many other antimicrobials^8^. SCVs of *S. aureus* are capable of evasion from iron sequestering mechanisms of the host. The SCV variants increase erythrocyte killing and upregulate high-affinity siderophores to uptake the released iron^9^. Also, *sigS* up regulation concomitant with H_2_O_2_ production leads to a reaction with intracellular iron (Fenton reaction), DNA damage, and cell death^10,11^. The Fenton reaction contributes to cell death induced by antibiotics in bacteria^12,13^.

Resistance to carbapenems is directed in three different ways, including increased expression of efflux systems, reduced porin expression, and overproduction of carbapenemases^14^. *Klebsiella pneumonia* carbapenemases (KPC)—acquired through transferable genes, are classified in class A of Ambler classification system. Moreover, overproduction of efflux pumps such as MexAB-OprM and reduced expression of porins including OprD lead to resistance to carbapenems^15–17^.

Vancomycin is an essential treatment for staphylococcal infections. Resistance to this drug becomes a critical issue in recent years^18^. Vancomycin-resistant and vancomycin-intermediate *S. aureus* (VRSA and VISA) are controlled through the *vanA* gene transferred on plasmid and missense mutations in *walk/R* genes, respectively^19,20^. In addition, fluoroquinolones are a suitable choice as treatment of staphylococcal infections. *S. aureus* resists against this class of antibiotics through over-expression of efflux pumps (*norA* gene) and mutation in topoisomerase IV encoded by *gyrA/B* and *grlA/B*^21,22^.

It has been reported that iron metabolism plays a central role in antibiotic resistance in *Escherichia coli*^23^; however, the relationship between iron concentration and antibiotic resistance in polymicrobial infections is unclear. Moreover, the role that ECF sigma factors play in antibiotic resistance is vague.

Therefore, this study aimed to determine how the iron-related ECF sigma factors would alter during *S. aureus* and *P. aeruginosa* interaction and how this alteration influenced the antibiotic resistance of persisters and wild-type isolates.

## Results:

### Coexistence of *S. aureus* and *P. aeruginosa*: inhibitory or stimulatory effect

**Figure 1:**
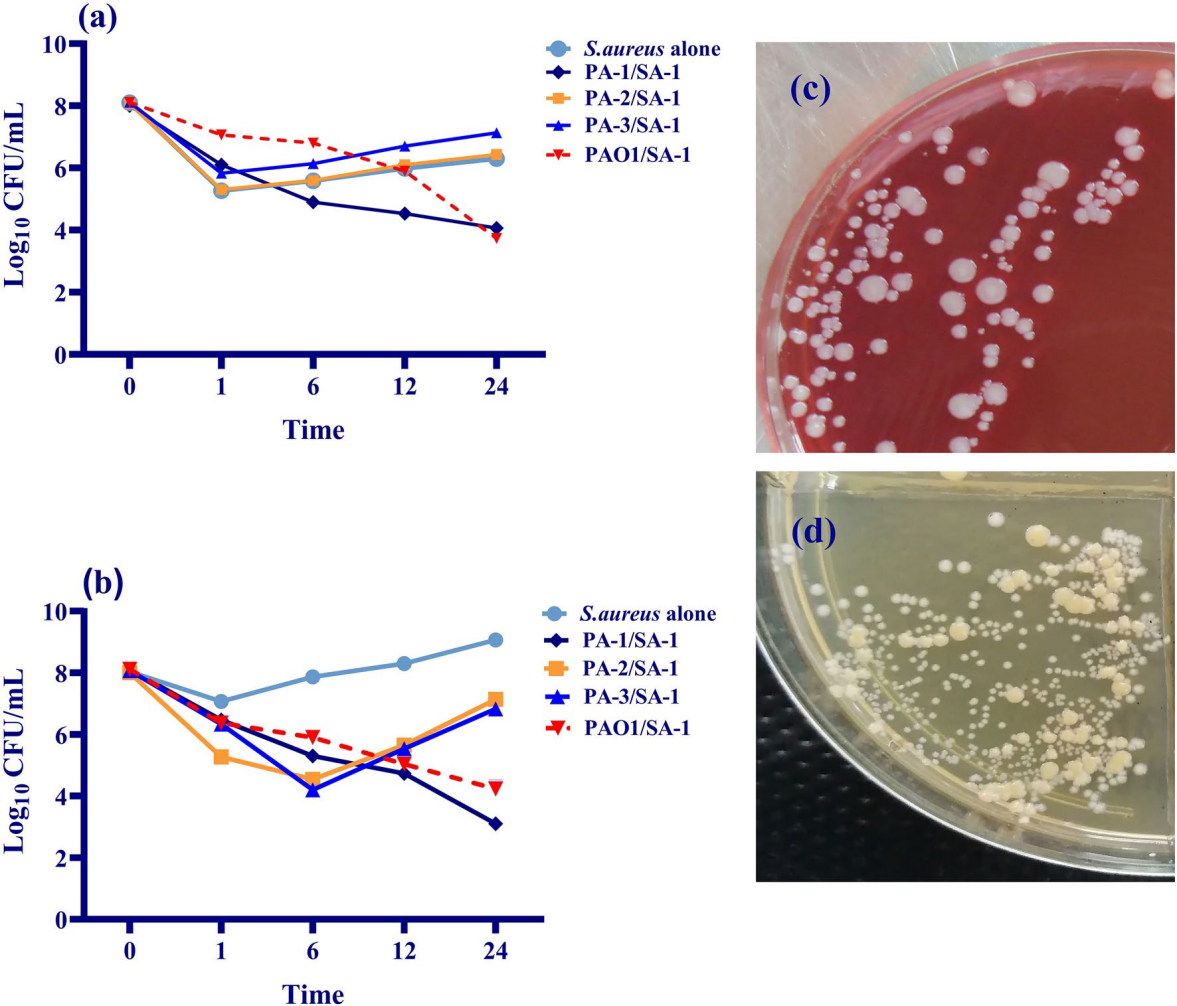
Co-culture evaluation of *S. aureus* and four strains of *P. aeruginosa* on the L929 cell line. The recovered strains from co-culture were determined as log_10_ CFU/mL counts. (**a**) The slow-growing colonies of *S. aureus* recovered from co-culture with different strains of *P. aeruginosa* in the planktonic state. (**b**) The slow-growing colonies of *S. aureus* recovered from co-culture with different strains of *P. aeruginosa* in the biofilm state. The inhibitory effect was observed on *S. aureus* viability in SA-1/PA-1 and SA-1/PAO1 co-cultures. In contrast, the co-culture of SA-1 with PA-2 and PA-3 strains leads to a stimulatory effect on viability. (**c**) Normal and slow-growing isolates of *S. aureus* on Colombia blood agar. (**d)** Normal and slow-growing isolates of *S. aureus* on BHI agar. Error bars indicate standard errors of the means from a representative triplicate assay.

A cell line-based model was developed to investigate the viability of *S. aureus* (SA-1) in co-culture with four clinical strains of *P. aeruginosa* (PA-1, PA-2, PA-3, and PAO1) in wound infections (Table 3). As illustrated in Fig. 1, *P. aeruginosa* caused a significant decrease in the viable colony counts of *S. aureus* recovered from the planktonic and biofilm states compared to monoculture. During the first hour of co-culture, a reduction in viability was detected for *S. aureus*, which was more remarkable in SA-1/PA-1 and SA-1/PAO1 co-cultures. The *S. aureus* viability in SA-1/PA-1 and SA-1/PAO1 in the planktonic and biofilm states decreased to approximately half of the monoculture (Fig. 1a,b). PA-1 belongs to sequence type 111(ST-111), a high-risk clone producing different toxins and phenazines. Compared to PA-2 and PA-3 (belong to ST-235), a more remarkable killing effect was observed in the combination of SA-1/PA-1.

In contrast, a stimulatory effect was observed in the co-culture of PA-2 and *S. aureus*. To clarify, the viable colony counts of *S. aureus* in SA-1/PA-2 co-culture almost reached the monoculture state (Fig. 1a,b). Interestingly, no significant decrease in viability was detected in *S. aureus* when co-cultured with PA-3 in both planktonic and biofilm conditions. In comparison to the planktonic state, the viability of *S. aureus* more notably decreased in the biofilm condition.

**Figure 2:**
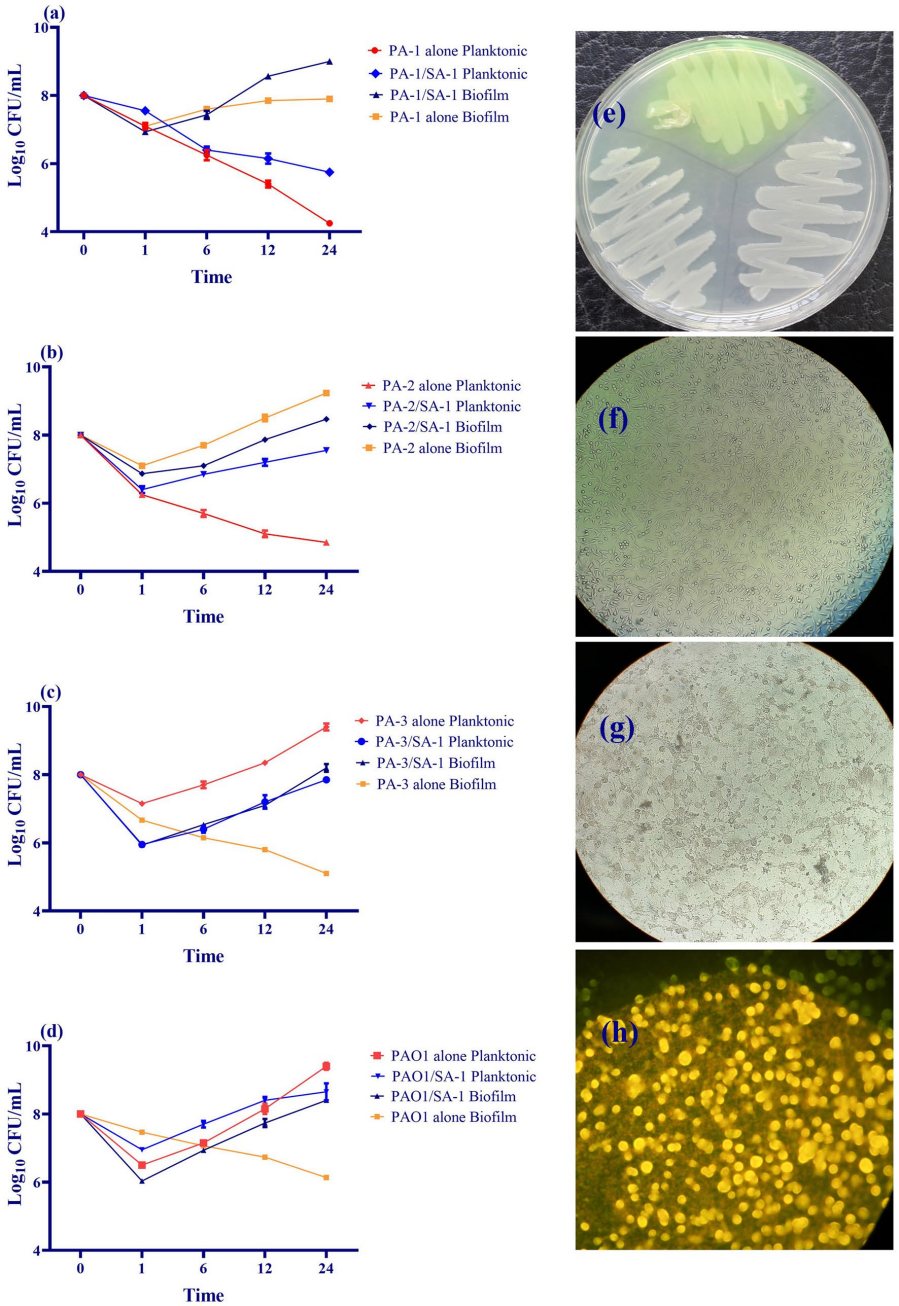
Co-culture evaluation of four strains of *P. aeruginosa* and *S. aureus* on the L929 cell line. The recovered strains from co-culture were determined as log10 CFU/mL counts. (**a**) The viable colony counts of PA-1 recovered from the planktonic and biofilm conditions in monoculture and co-culture with SA-1. (**b**) The viable colony counts of PA-2 recovered from the planktonic and biofilm conditions in monoculture and co-culture with SA-1. (**c**) The viable colony counts of PA-3 recovered from the planktonic and biofilm states in monoculture and co-culture with SA-1. (**d**) The viable colony counts of PAO1 recovered from the planktonic and biofilm conditions in monoculture and co-culture with SA-1. (**e**) The phenazine production was inhibited during co-culture with SA-1. PA-1 produced green pigment before co-culture with SA-1, while the pigment production was inhibited during the co-culture with SA-1. (**f**) Monolayer of L929 cell line was captured by inverted microscope (Olympus, BioTek, VT, USA). (**g**) Monolayer of L929 cell line infected by *S. aureus* and *P. aeruginosa* was captured by inverted microscope (Olympus, BioTek, VT, USA). h) Monolayer of L929 cell line infected by *S. aureus* and *P. aeruginosa*, stained by Propidium Iodide (Sigma, USA) and acridine orange (Sigma, USA), and was captured by an olympus fluorescence microscope (Olympus, BioTek, VT, USA). Error bars indicate standard errors of the means from a representative triplicate assay.

As depicted in Fig. 2, the viability of PA-1 and PAO1 did not change compared to the monoculture. The viable colony counts in both biofilm and planktonic forms indicated a negligible effect of *S. aureus* on *P. aeruginosa* viability. Contrary to PA-1/SA-1 and PAO1/SA-1, the viability of PA-2 and PA-3 reduced in comparison to monoculture. Remarkably, following co-culture with *S. aureus*, the recovered *P. aeruginosa* strains demonstrated a highly mucoid phenotype in which the phenazine-producing strains converted to a non-phenazine producer.

### *S. aureus* strain converted to a slow-growing phenotype during co-culture

Notably, following the co-culture with *P. aeruginosa*, the growth rate of *S. aureus* colonies reduced significantly. Although *P. aeruginosa* strains grew naturally at 37 °C and in ambient air after overnight incubation, *S. aureus* indicated a slow growth. In other words, slow-growing, tiny, non-hemolytic colonies of *S. aureus* were recovered five days post-plating. The diluted samples were plated on Columbia agar supplemented with 5% sheep blood and incubated at 37 °C and 5% CO_2_, while the monoculture of *S. aureus* was recovered typically at 37 °C and ambient air after 18–24 h. Also, mannitol consumption was restrained in these phenotypes during the growth on MSA. Also, auxotrophy to menadione, thymine, and hemin was investigated on the persister strains, but no auxotrophy was detected.

### The activity of iron regulating ECF sigma factors altered during co-culture

#### Iron regulating ECF sigma factors of *P. aeruginosa*

**Figure 3:**
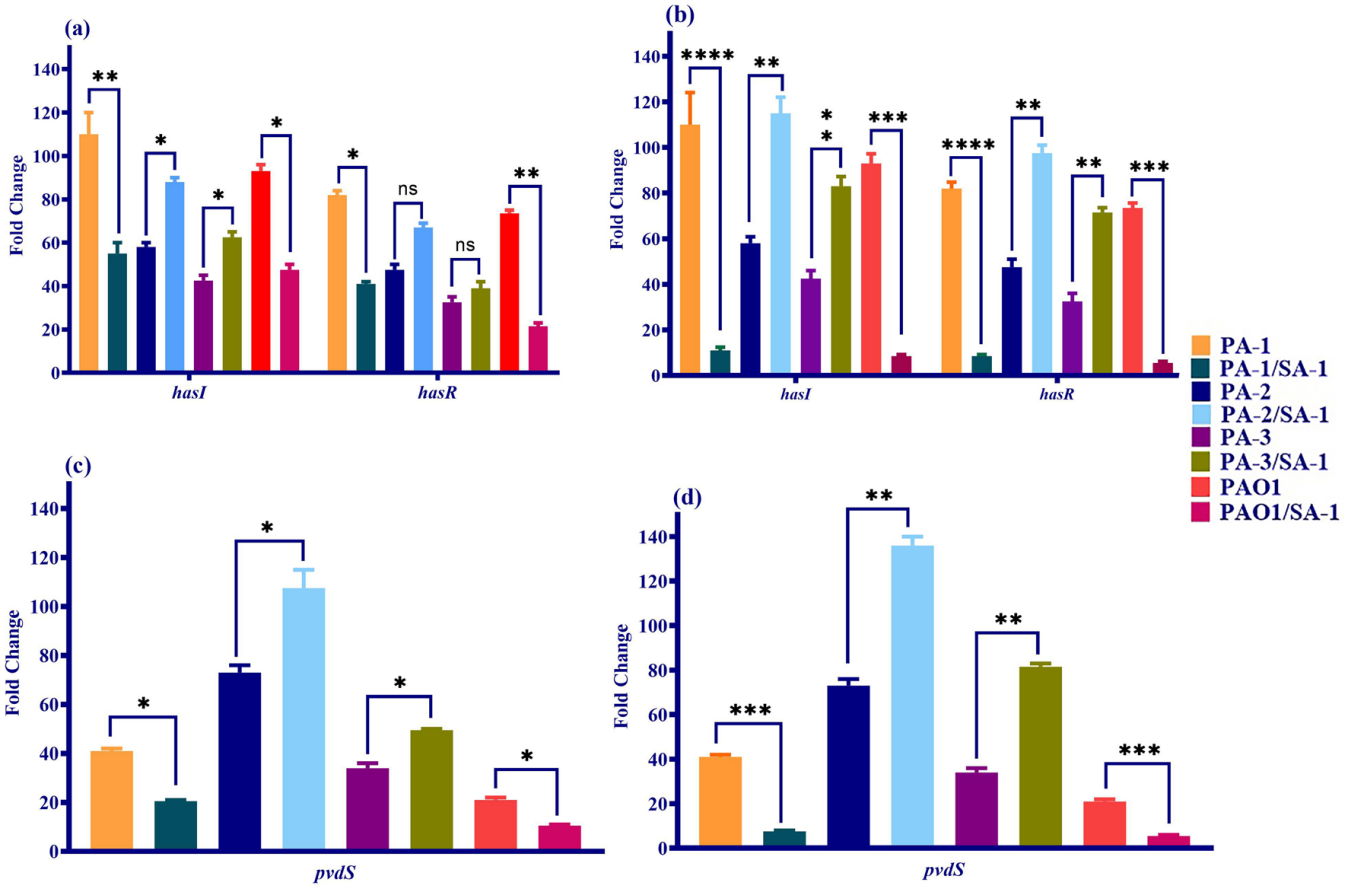
The expression level of ECF sigma factors of *P. aeruginosa* in different states of co-culture. (**a**) The expression level of *hasI* and *hasR* in the planktonic co-culture compared to the monoculture and control strain. (**b**) The expression level of *hasI* and *hasR* in the biofilm state of co-culture in comparison to the monoculture and control strain. (**c**) The *pvdS* changes in *P. aeruginosa* strains in the planktonic forms of the co- and monoculture. (**d**) The *pvdS* changes in *P. aeruginosa* strains in the biofilm forms of the co- and monoculture. Each data set was analyzed using the student's t-test, and the Holm-Sidak method for multiple comparison. The data were presented as Mean + SEM. *p-value < 0.05; **p-value < 0.01; ***p-value < 0.001; ****p-value < 0.0001.

Iron is tightly regulated in co-culture conditions due to its critical role in the adaptation of *S. aureus* and *P. aeruginosa*. Therefore, the ECF sigma factors were investigated in a co-culture model. As illustrated in Fig. 3, the expression level of *hasI* and *pvdS* decreased significantly in PA-1/SA-1 and PAO1/SA-1 combinations compared to PA-1 and PAO1 monocultures. Meanwhile, the expression level of *hasR* (a receptor gene to sigma factor *hasI*) and pyoverdine production reduced remarkably. Although *hasI* downregulated to two-fold in the planktonic state, a more than ten-fold decrease was observed in the biofilm state in PA-1 and PAO1. A similar downregulation (a two- and five-fold decrease in the planktonic and the biofilm states, respectively) was observed in *pvdS*, which lead to a reduction in pyoverdine production in the two mentioned strains. In contrast, *pvdS* and *hasI* expression levels increased to approximately ninety percent in the biofilm state, whereas a half increase was detected in the planktonic conditions of PA-2 and PA-3.

The siderophore production and ECFs downregulation occurred in consistence to increased killing of *S. aureus* by PA-1 and PAO1 (Figs. 3 and 5a). During the co-culture of PA-1 and PAO1 with *S. aureus*, pyoverdine production decreased compared to monoculture. Also, *hasR* downregulated due to iron boost in the co-culture media (Fig. 3a,b). Conversely, the pyoverdine production indicated a non-significant increase in PA-2 and PA-3 in comparison to monoculture conditions and the wild-type strain (Fig. 5a). Compared to the monoculture findings and gene expression level in *P. aeruginosa* strains in iron-rich and -starved media, the remains of *S. aureus* were used as iron sources by *P. aeruginosa*.

#### Iron regulating ECF sigma factor of *S. aureus*

*sigS* is the only known ECF sigma factor in *S. aureus*. Contrary to *P. aeruginosa*, the role of *sigS* in iron regulation has not been investigated; however, it may play a role in Fenton's reaction through iron regulation. Therefore, the expression level of *sigS* was observed in the co-culture of *S. aureus* with different strains of *P. aeruginosa.*

**Figure 4:**
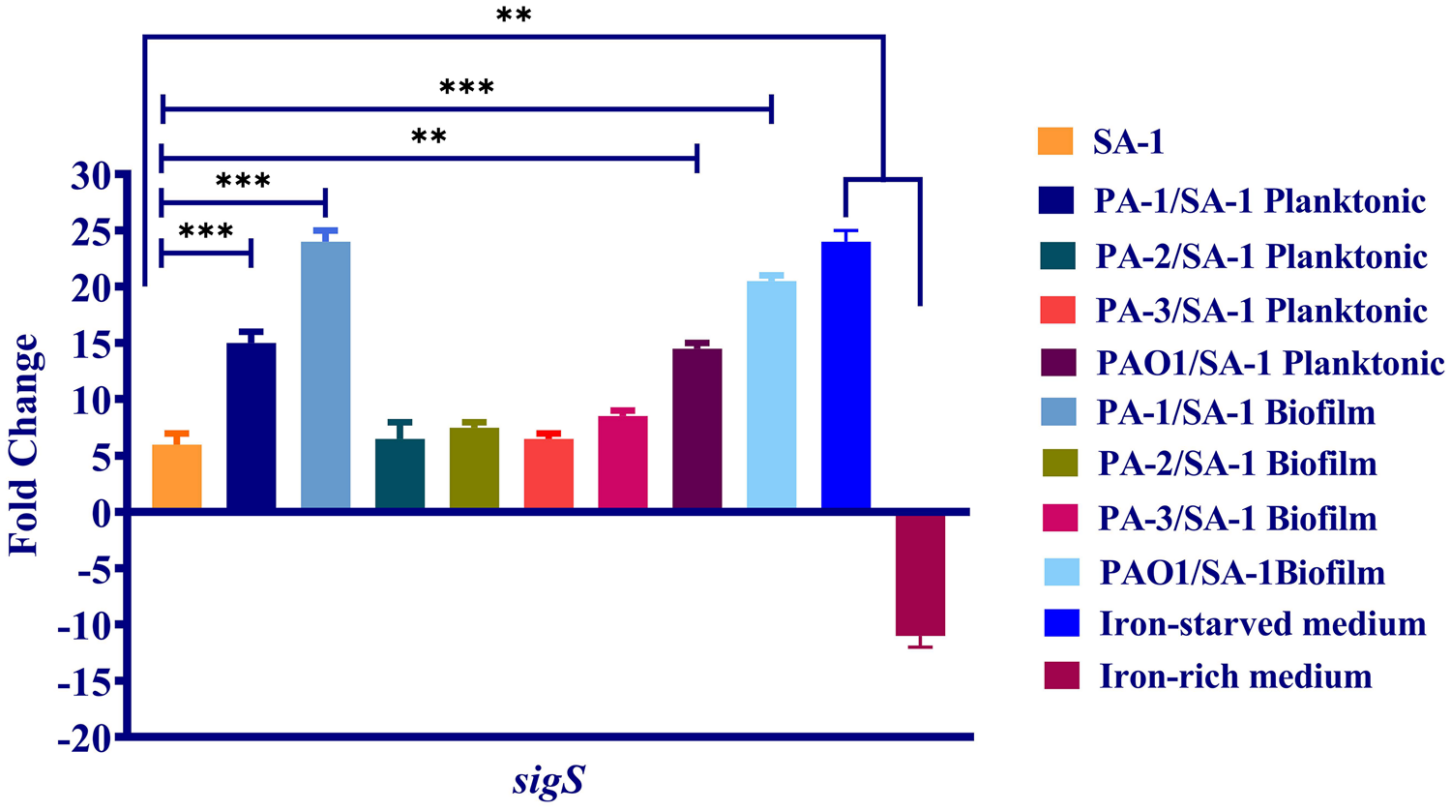
The expression level of ECF sigma factors of *S. aureus* in different states of co-culture. The changes in the *sigS* expression level of slow-growing phenotypes of *S. aureus* in the planktonic and biofilm state of co-culture compared to monoculture. Also, the expression level of *sigS* in the iron-rich and iron-starved medium. Each data set was analyzed using the student's t-test, and the Holm-Sidak method for multiple comparison. The data were presented as Mean + SEM. *p-value < 0.05; **p-value < 0.01; ***p-value < 0.001; ****p-value < 0.0001.

As depicted in Figs. 4, *sigS* upregulated in the co-culture with PA-1 and PAO1. The expression level of *sigS* increased two- and three-fold in the planktonic and biofilm forms compared to monoculture. Interestingly, the siderophore production decreased in the co-culture with PA-1 and PAO1 in both planktonic and biofilm states. Regarding the considerable killing effect of the two mentioned strains on *S. aureus* and slow-growing isolates recovered from co-culture, it seems that the iron starvation leads to *sigS* upregulation. Therefore, the expression level of *sigS* was investigated in iron-rich (> 1 µM) and -starved (< 0.5 µM) media, and the findings mentioned above were confirmed (Fig. 4).

**Figure 5:**
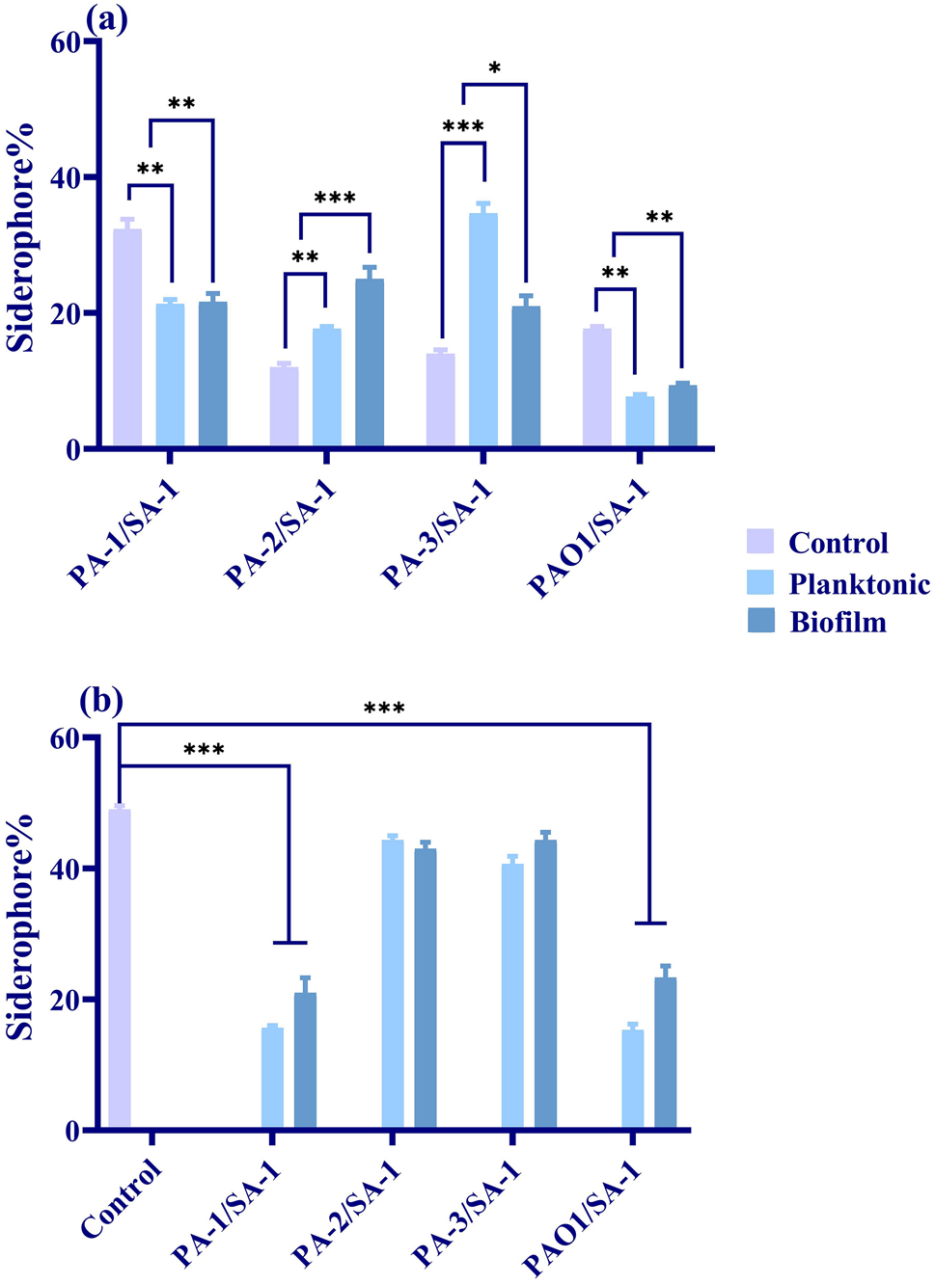
Siderophore production (**a**) *P. aeruginosa* and (**b**) *S. aureus* in the monoculture, the planktonic and biofilm states of co-culture. Each data set was analyzed using the student's t-test, and the Holm-Sidak method for multiple comparison. The data were presented as Mean + SEM. *p-value < 0.05; **p-value < 0.01; ***p-value < 0.001; ****p-value < 0.0001.

Despite the upregulation of *sigS* in the co-culture with PA-1 and PAO1, a slight increase was observed in SA-1/PA-2 and SA-1/PA-3. Moreover, the siderophore production indicated a non-remarkable decrease in the SA-1/PA-2 and SA-1/PA-3 combinations compared to monocultures (Fig. 5b). Although a coexistence relationship was observed between SA-1 and PA-2 and PA-3, the expression level of *sigS* increased and recovered isolates of *S. aureus* slowly grew. Consider the significant increase of siderophores in co-culture with PA-2 and PA-3 in the iron-rich medium; it is suggested that despite coexistence, SA-1 competes for nutrients with the biofilm-forming and susceptible strains of *P. aeruginosa*.

### Antibiotic resistance had a relationship with ECF sigma factors

Iron limitation affects some genes encoding antibiotic resistance. Since iron competition between *S. aureus* and *P. aeruginosa,* we examined the hypothesis of whether the antibiotic resistance is influenced by co-culture or not. Changes of different antibiotics categories in *P. aeruginosa* were listed in Table1.

**Table 1:** Antibiotic susceptibility of recovered *P. aeruginosa* strains.

Antibiotics	Wild type				Planktonic state of co-culture				Biofilm state of co-culture			
	PA-1	PA-2	PA-3	PAO1	PA-1/SA-1	PA-2/SA-1	PA-3/SA-1	PAO1/SA-1	PA-1/SA-1	PA-2/SA-1	PA-3/SA-1	PAO1/SA-1
Imipenem	16	16	2	0.5	2	2***	1	8	2	8***	4	8
Meropenem	64	32	16	0.5	8***	16	16***	16***	8***	8	64***	16***
Doripenem	64	32	1	0.5	8*	16*	8*	4**	8*	16*	16*	4**
Amikacin	16	32	2	0.5	4***	4***	4*	3*	8**	4***	3***	4***
Ciprofloxacin	64	16	0.5	0.5	32*	64**	16***	16***	32*	32*	16***	16***

*P-value < 0.01; **P-value < 0.001; ***P-value < 0.0001.

**Figure 6:**
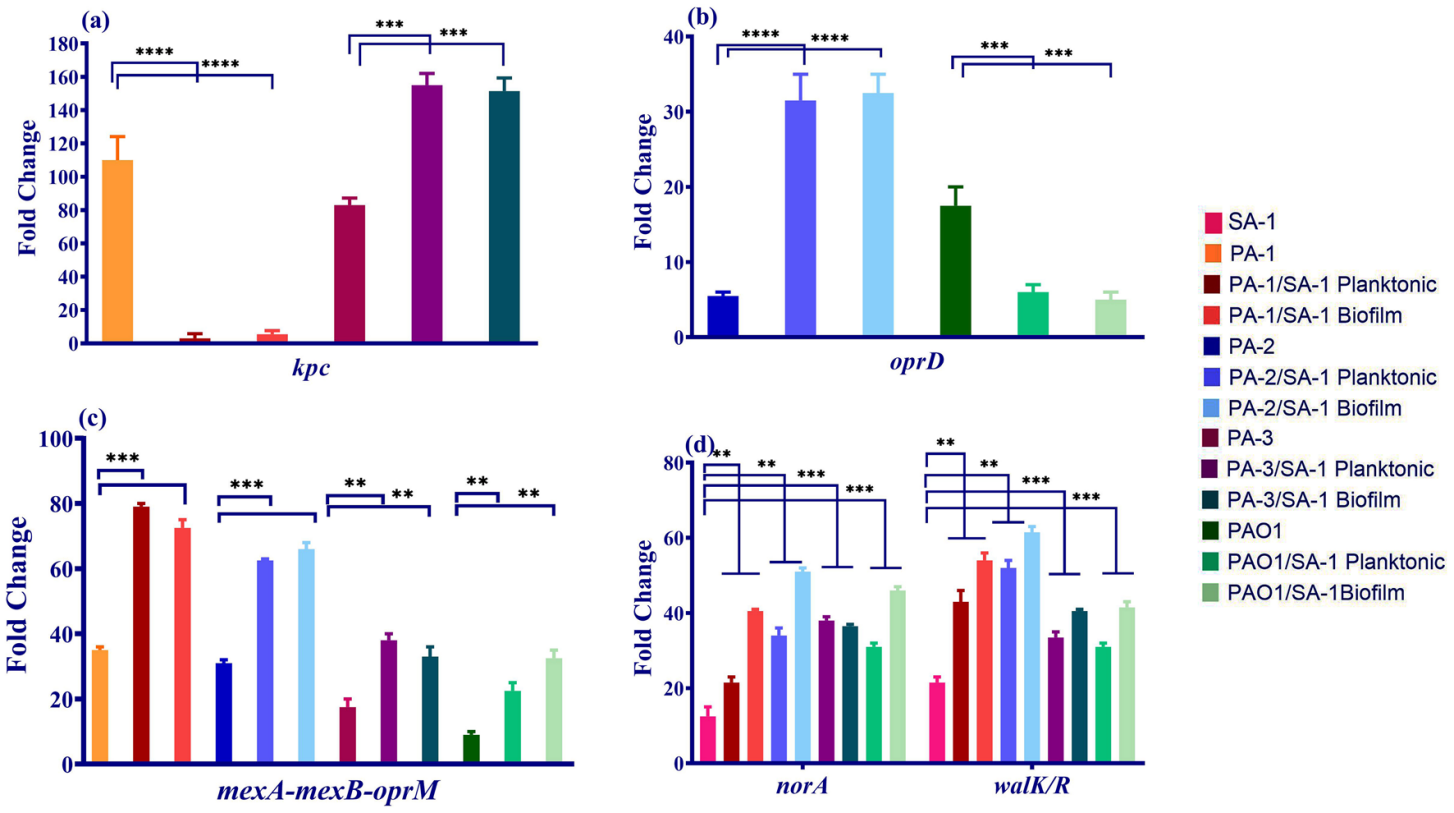
The expression level of resistance genes in *P. aeruginosa* and *S. aureus* in different states of co-culture. (**a**) The expression level of *kpc* in the planktonic and biofilm states of co-culture compared to the monoculture. (**b**) The expression level of *oprD* in the planktonic and biofilm states of co-culture compared to the monoculture (**c**) The *mexA-mexB-oprM* changes in *P. aeruginosa* strains in the planktonic and biofilm forms of the co- and monoculture. (**d**) The *norA* and *walk/R* changes in *S. aureus* strains in the planktonic and biofilm forms of the co- and monoculture. Each data set was analyzed using the student's t-test, and the Holm-Sidak method for multiple comparison. The data were presented as Mean + SEM. * p-value < 0.05; **p-value < 0.01; ***p-value < 0.001; ****p-value < 0.0001.

Carbapenems, including imipenem, meropenem, and doripenem, showed various changes after co-culture with *S. aureus.* To illustrate, the MIC of meropenem and doripenem increased in the PA-1 and PAO1, while the imipenem resistance level decreased remarkably in PA-1 (MIC: 2 µg/mL) and increased in PAO1. The expression level of genes encoding resistance to carbapenems, including *kpc*, efflux pumps (*mexA-mexB-oprM*), and *oprD* was investigated to indicate the reason for this contradiction. The expression level of *kpc* decreased more than 100-fold in PA-1, whereas *mexA-mexB-oprM* upregulated (Fig. 6a,c). As well, *oprD* downregulated in PAO1 (Fig. 6b). Unlike PAO1, *oprD* upregulated in PA-2, while the expression level of *kpc* upsurged in PA-3 (Fig. 6). The MIC of carbapenems decreased in PA-2, whereas a significant increase was detected in PA-3.

The correlation between ECFs and antibiotic resistance was investigated, and as demonstrated in Table 1, there is a strain-dependent relationship between the expression level of ECFs and *kpc*, *mexA-mexB-oprM,* and *oprD*. In other words, a significant direct relationship was observed between *kpc* downregulation, *oprD* upregulation, and ECFs' decreased expression level. No association was identified between ECFs and resistance to meropenem and ciprofloxacin regarding the high expression level of *mexA-mexB-oprM* and the MIC amounts in all strains.

In addition, the resistance level was examined in an iron-rich media. As the medium was supplemented with iron, a similar reaction was observed in SA-1/PA-2 and SA-1/PA-3 combinations, too. Likewise, the apparent relationship between ECF and *kpc* downregulation was detected in monocultures of *P. aeruginosa* in the iron-rich medium. Interestingly, the viability of *S. aureus* augmented two-fold in combination with PA-1 and PAO1 in the iron-rich medium.

As mentioned above, *S. aureus* strain converted to a slow-growing phenotype. The antibiotic susceptibility was investigated in this phenotype. As shown in Table 2, the MIC of ciprofloxacin increased from 0.5 to 64 µg/mL after co-culture with all four strains of *P. aeruginosa.* The expression level of *norA* as an efflux pump involves resistance to ciprofloxacin was checked. According to Fig. 6, *norA* upregulated in *S. aureus* after co-culture. However, no changes were detected in the expression level of *gyrA/B* and *grlA/B* in comparison to monoculture.

**Table 2:** Antibiotic susceptibility of recovered *S. aureus* strains.

Antibiotics	Wild type	Planktonic state of co-culture				Biofilm state of co-culture			
	SA-1	PA-1/SA-1	PA-2/SA-1	PA-3/SA-1	PAO1/SA-1	PA-1/SA-1	PA-2/SA-1	PA-3/SA-1	PAO1/SA-1
Vancomycin	0.25	256***	256***	256***	256***	256***	256***	256***	256***
Ciprofloxacin	0.5	16*	8	16*	16*	32**	32**	64**	8
Gatifloxacin	16	8*	4**	8*	2***	4**	8*	2***	2***

* P-value < 0.01; **P-value < 0.001; ***P-value < 0.0001.

NorA overproduction and resistance to ciprofloxacin were observed in both iron-rich and -starved media; however, to a lesser extent in the former. Unlike the co-culture conditions, *norA* was overexpressed in the monocultures in the iron-starved medium, while such reaction was not detected in monocultures of the iron-rich medium.

Moreover, a notable resistance to vancomycin was detected in *S. aureus* when co-culture with *P. aeruginosa* strains. The MIC of vancomycin upsurged from 0.5 to 256 µg/mL after co-culture. The mechanisms for vancomycin resistance, including *vanA*, *vanS*, and *walk/R,* were investigated to determine the reason for the MIC escalation. SA-1 did not possess the *vanA* gene and *van* operon as examined by the PCR method. As depicted in Fig. 6, the expression level for *walk/R* increased significantly during co-culture compared to monoculture.

Resistance to vancomycin in SA-1 was studied in the iron supplemented medium, and a heterogeneous population of *S. aureus* was recovered from co-culture. Some vancomycin-sensitive isolates grew normally after 18–24 h, while some isolates slowly recovered after five days and were completely resistant to vancomycin. Although a slight increase in *sigS* expression level was detected in the first group, a remarkable upsurge in the expression of *sigS* and *walk/R* was observed in the second group in comparison to monoculture.

The correlation between *sigS* and resistance to ciprofloxacin and vancomycin was explored. According to Fig. 6 and Table 2, a significant direct relationship was noticed between *sigS* and vancomycin resistance due to *walk/R* overexpression. Also, *norA* overexpression and *sigS* expression levels were increased consistently and indicated a significant statistical association.

## Discussion

The essential content of iron in bacteria is 10^–7^–10^–5^ M; however, the host maintains its extracellular iron content as 10^–8^ M. Therefore, it is necessitated for the pathogens to acquire iron using different mechanisms, including siderophores, heme assimilation systems, and ferric iron uptake systems^24^. During coinfection, the competition for micronutrients, including iron, determines the type of relationship between *S. aureus* and *P. aeruginosa*. Moreover, *S. aureus* aggregates and attaches to the cell substratum and initiates the biofilm formation process. The early biofilm formed by *S. aureus* contributes to *P. aeruginosa* to co-aggregation and development of dual-species biofilms^25^. As depicted in Figs. 1 and 2, the viability of *S. aureus* varied in co-culture with different strains of *P. aeruginosa*. As SA-1 co-cultured with PA-1 (ST111) and PAO1, the viability reduced to half of the monoculture. During co-culture, *P. aeruginosa* secrets LasA protease and HQNO (2-heptyl-4-hydroxyquinoline-N-oxide) to kill *S. aureus* and obtain iron using siderophores^26,27^. LasA protease production increased in *P. aeruginosa* strains recovered from co-culture compared to the monocultures (data not shown). Moreover, the pyoverdine production dramatically decreased in PA-1 and PAO1 as co-cultured with SA-1 (Fig. 5). *P. aeruginosa* and *S. aureus* co-infection provide an iron-rich environment for the former, leading to decreased pyoverdine production^28^. Regarding the decreased expression level of *pvdS* and *hasI*, it is suggested that PA-1 and PAO1 used SA-1 as an iron source. Mashburn and et al. reported that during co-culture with *S. aureus*, *P. aeruginosa* downregulated the iron uptake genes due to the presence of a sustainable source of iron^28^. The ECF sigma factors play different roles in the survival and fitness costs of the bacteria. As iron sources decreased in the environment, the anti-sigma factor protein degrades, therefore the expression levels of *hasI* and *pvdS* increase. Consequently, the production of HasR (a corresponding receptor of *hasI*) and pyoverdine would be augmented^29,30^. The ECF downregulation, HasR and pyoverdine decrease in the co-culture of *S. aureus* with *P. aeruginosa* were observed in the iron-rich medium.

Contrary to the high killing ability of PA-1 and PAO1, no significant decrease in viability was observed in SA-1 co-cultured with PA-2 and PA-3. PA-1 strain belonged to ST-111, while PA-2 and PA-3 belonged to ST-235. Although both STs are hypervirulent, high-risk clones, it is suggested that the characteristics of the strains, including toxin production, played an essential role in the relationship of SA-1 with PA-2 and PA-3^31,32^. As demonstrated in the results section, PA-2 and PA-3 indicated a negligible effect on SA-1 viability, and also ECFs downregulated. The pyoverdine and HasR production increased slightly. The viability of SA-1 in the co-culture with PA-2 and PA-3 did not differ significantly in the iron-rich and -starved media. Therefore, it seems a strain-dependent killing behavior caused the coexistence of SA-1 with PA-2 and PA-3.

ECFs play various roles in antibiotic resistance, virulence, and metabolism. The relationship between antibiotic resistance and *P. aeruginosa* ECFs was investigated during co-culture with *S. aureus*. Moreover, genes encoding resistance to carbapenems were investigated. Resistance to carbapenems often occurs due to carbapenemases (encoding by *kpc*) preferably against imipenem, porins (encoding by *oprD*) against imipenem and doripenem, and efflux pumps (*maxA-mexB-oprM*) against meropenem. As depicted in Fig. 3, the expression level of *hasI* and *pvdS* decreased in staphylolytic strains of *P. aeruginosa*. Iron boost in the environment due to the lysis of *S. aurues* led to a decrease in *kpc* expression level, while *maxA-mexB-oprM* and *oprD* were overexpressed. Imipenem resistance preferably occurs due to the loss of *oprD* and carbapenemases (enzymes encoding by *kpc*), whereas *maxA-mexB-oprM* efflux pumps cause resistance to meropenem. PA-1 and PA-3 strains possess the *kpc* gene, and after co-culture with *S. aureus,* a significant decrease in *kpc* expression level was observed in PA-1 while PA-3 showed upregulation. The MIC of imipenem in PA-1 and PA-3 confirmed that iron limitation leads to *kpc* upregulation. At the same time, *maxA-mexB-oprM* upregulated to tenfold in PA-1. Also, the expression level of *maxA-mexB-oprM* increased in PAO1. MIC of meropenem increased from 32 and 0.5 to 64 for PA-1 and PAO1. PA-2 does not possess *kpc* gene, but a significant decrease in the carbapenems’ MIC was detected in this strain. The expression level of *oprD* as an import gate for carbapenems increased remarkably due to iron limitation. Unlike previous reports that described a direct relationship between the iron limitation and *maxA-mexB-oprM* overexpression, our findings indicated that *S. aureus* beyond the role as an iron source might affect the regulation of antibiotic resistance^33^. Moreover, the MIC of ciprofloxacin elevated from 16 to 512 in consistency with *maxA-mexB-oprM* overexpression. As reported by Ankley, the iron chelation lead to a decrease in resistance to antimicrobial compounds^34^. Moreover, iron plays an important role in the early stages of biofilm formation, which indirectly may influence antibiotic resistance^35^. Overexpression of ferric reductase leads to antibiotic mediated cell death through the Fenton reaction in *P. aeruginosa*. Such an effect was induced abundantly in exposure to gentamycin, norfloxacin, tetracycline, and ampicillin^13^.

*sigS* as the only ECF identified in *S. aureus* plays different roles in survival during starvation, stresses caused by DNA and cell wall damages, and oxidative stresses^36^. The expression level of *sigS* increased during the co-culture with *P. aeruginosa* strains. Although the survival of SA-1 in combinations with PA-2 and PA-3 were not influenced extensively, the metabolism and growth were affected. The recovered isolates of SA-1 after co-culture slowly grew and lost the ability for hemolysis, mannitol consumption, and pigment production. Different studies reported that *P. aeruginosa* lyses *S. aureus* during co-culture to provide iron and nutrient to grow. Moreover, *P. aeruginosa* acts as a stress agent for *S. aureus* and leads it to persistence^25,37^. According to Fig. 4, the siderophore production decreased in co-culture with *P. aeruginosa* strains, particularly PA-1 and PAO1. Moreover, *sigS* expression level reduced in SA-1 co-cultured with *P. aeruginosa* in an iron-rich medium.

Based on Table 2, the MIC of ciprofloxacin increased from 0.5 to 8 µg/mL in SA-1 and *norA* as an encoding gene for NorA efflux pump, overexpressed in both co-culture states. Iron limitation leads to *norA* overexpression and increased resistance to ciprofloxacin in *S. aureus*^38–40^. The overexpression of *norA* occurs due to the *mgrA* regulatory effect, which causes resistance to ciprofloxacin and resistance to vancomycin through an unknown mechanism^41^. *sigS* expression contributes to *S. aureus* to persist in starvation condition and confer resistance against DNA damages^42^.

Interestingly, the slow-growing isolates of *S. aureus* remarkably became resistant to vancomycin. The MIC of vancomycin upsurged from 0.5 to 512 µg/mL after co-culture. Although SA-1 did not possess *van* operon, the expression level of *walk/R* increased significantly after co-culture with *P. aeruginosa*. Reduced vancomycin susceptibility occurs due to inactivation of *walk/R* two-component system, responsible for cell wall synthesis in *S. aureus*^43,44^. As Miller and et al. reported, *sigS* mutants were more susceptible to cell-wall targeting antibiotics and DNA damaging agents, including ciprofloxacin; it is suggested that *sigS* overexpression in co-culture with *P. aeruginosa* is related to *walk/R* inactivation and decreased sensitivity to vancomycin^42^. Also, *sigS* upregulation concomitant with H_2_O_2_ production leads to a reaction with intracellular iron (Fenton reaction), DNA damage, and cell death^10,11^. The Fenton reaction contributes to cell death induced by antibiotics in bacteria^12,13^. Oxidative stress might play a role in resistance to ciprofloxacin in *ΔkatA* (mutation in genes encoding catalase) mutants; and therefore, the iron assimilation genes would be silenced to defend bacteria against oxidative stress^45^.

Iron as a critical factor regulates resistance to different antibiotics. During co-culture, iron metabolism altered because of competition between *P. aeruginosa* and *S. aureus*, resulting in changes in antibiotic resistance. The ECF sigma factors play a role in regulating iron and, consequently, influence antibiotic resistance of the infection strains. Although a direct relationship between *sigS* and antibiotic resistance was observed in the current study, it is necessary to conduct more studies on the exact association between resistance and ECF sigma factor in *S. aureus* during co-culture.

## Materials and methods

### Study design

One clinical strain of *S. aureus* and three clinical strains of *P. aeruginosa* were selected based on their characteristics, mentioned in Table 3. The strains were obtained from the microbial bank of the Microbiology Laboratory of Hamadan University of Medical Sciences. The clinical strains were chosen regarding their characteristics, including biofilm formation, toxin production (T1SS, T2SS, and T3SS secretion systems for *P. aeruginosa*, Panton-Valentine Leukocidin (PVL) and alpha toxins for *S. aureus*), iron-related sigma factors, and antibiotic susceptibility. Also, the clinical strains of *P. aeruginosa* were chosen regarding their phenotypic and genotypic characteristics (based on molecular detection of virulence and resistance genes, quantitative measurement of virulence production, and resistance according to CLSI guidelines) to examine the influence of particular properties of different strains in co-culture. *Staphylococcus aureus* ATCC25923 and *Pseudomonas aeruginosa* PAO1 were used as the control strains. This study was conducted under the ethical approval code IR.UMSHA.REC.1399.129.

**Table 3:** Strains used in this study.

Strains	Species	Source	Sequence type	Characteristics	
				Virulence	Antibiotic susceptibility
PA-1	*P. aeruginosa*	Wound	111	Toxin-producing strain^1^/Pyoverdine Producer	MDR strain
PA-2	*P. aeruginosa*	Wound	235	Biofilm-forming strain	MDR strain
PA-3	*P. aeruginosa*	Wound	235	Non biofilm- former/Non toxin- producer	Susceptible strain
PAO1	*P. aeruginosa* PAO1	Standard Strain	–	Biofilm-forming strain/Toxin-producing strain	Susceptible strain
SA-1	*S. aureus*	Wound	5	PVL(Panton-Valentine Leukocidin)^2^ producing/biofilm-forming strain/Siderophore producer/	MDR strain
Control strain	*S. aureus* ATCC25923	ATCC strain	243	Non biofilm- former/ PVL producing	Susceptible strain

^1^Toxin-producing strain: Strains which produced at least 3 toxins of Secretion systems I, II, and III.

^2^Panton-Valentine Leukocidin (PVL) is a membrane-targeting toxin of virulent strains of *S. aureus.*

### Growth condition

Trypticase soy broth (TSB) (Merck, Germany), mannitol salt agar (MSA) (Merck, Germany), and cetrimide agar (CA) (Merck, Germany), Columbia agar (Merck, Germany) containing 5% sheep blood, and BHI (Merck, Germany) containing 6% NaCl were used as culture media to cultivate and recover the slow-growing phenotypes, *S. aureus*, and *P. aeruginosa* strains. The plates were incubated in both ambient air and 5% CO_2_, at 37 °C and 25 °C.

### Cell culture

To investigate the interaction of *S. aureus* and *P. aeruginosa* in wounds, the mouse fibroblast cell line (subcutaneous connective tissue)—L929 was obtained from the Pasteur Institute of Iran. The cell line was cultured as described in the study of Dehbashi et.al^46^. Briefly, It was cultured on in the high glucose DMEM medium (DNA BioTech, Iran) supplemented with 10% FBS (Invitrogen, USA) and penicillin–streptomycin (to a final concentration of 50–100 IU/mL for the former one and 50–100 µg/mL for the last one) (Sigma, USA). Then, the cell line was sub-cultured to 24-well plates for further investigations.

### Co-culture condition

Mono- and co-culture assessment of the bacteria were done on L929 monolayer based on Dehbashi et al.^8^. Briefly, the monolayer of L929 was prepared in 24-wells cell culture plates. Then, the DMEM medium was removed, and the bacterial cultures (in exponential phase) were washed in PBS. 100 µL of bacterial suspension with OD_600_:0.1 in 1 mL MEM containing l-Glutamine was added to the wells as co-culture (each *S. aureus* and *P. aeruginosa* strain in each well). The plates were incubated at 37 °C and 5% CO2. In 1, 6, 12, and 24 h intervals, the media were aspirated, diluted, and plated on MSA and CA. Then the fresh medium was added. After 24 h incubation, to recover the *S. aureus* and *P. aeruginosa* strains, the planktonic bacteria were diluted in fresh PBS and cultured on MSA, CA, BHI, and Columbia agar. Then, following twice washing with PBS, 200 µL of 0.1% Triton X-100 was added to each well, and gently agitated for 30 min. The cells were scraped by a cell scraper to disrupt the biofilm. Then, the bacteria were diluted and plated as described for the planktonic co-culture. Each experiment was done in triplicate.

### Antimicrobial susceptibility

The antimicrobial susceptibility of recovered strains of *S. aureus* and *P. aeruginosa* were tested based on CLSI 2019 for different categories of antibiotics, including beta-lactams, aminoglycosides, fluoroquinolones, and carbapenems using E-test strips (Liofilchem, Italy). The categories of antibiotics were selected based on the clinical guidelines of infections’ treatments. Moreover, the antimicrobial susceptibility of *S. aureus* and *P. aeruginosa* in the co-culture was investigated. Five concentrations of each antibiotic were added to the infected cell monolayer, and in 1, 6, 12, and 24 h intervals, the medium was replaced with fresh MEM + antibiotics, and then the samples were plated as described in the past section. All the tests were done in triplicate.

### Siderophore production

The spectrophotometry method based on El-fouly et al. was used to investigate siderophore production^46,47^. Briefly, *P. aeruginosa* strains were inoculated to RPMI1640 (Invitrogen, USA) and incubated at 37 °C by shaking 100 rpm overnight. The OD_600nm_ of the cultures was measured. After centrifugation at 200 g for 30 min, the supernatants were collected and filtered by 0.22 µm Millipore filters (Merck, Germany). The OD_405nm_ of supernatants was measured spectrophotometrically, and then Relative Pyoverdine Production (RPP) was calculated by the following formula: RPP: OD_405_/OD_600_.

The siderophore amount in *S. aureus* was measured using succinic acid broth, containing K_2_HPO_4_, KH_2_PO_4_, (NH_4_)_2_, MgSO_4_, succinic acid. The suspected strains were inoculated into the media and incubated at 30 °C for 24 h, shaking at 200 rpm. Then, the media was centrifuged at 10,000×*g* for 10 min. The supernatant's absorbance was measured at OD_400_ using a spectrophotometer^48^.

### RNA isolation and RT-PCR

The total RNAs isolation and cDNA synthesis were performed on bacteria during the co-cultures using the GeneAll extraction and cDNA synthesis kit (GeneAll, Korea) based on the manufacturer's instructions.

The gene expressions of *hasI*, *pvdS*, *sigS*, *kpc, oprD, mexA-mexB-oprM, norA,* and *walk/R* were analyzed using real-time PCR based on the primers listed in Table 4. Based on previous studies, *nucA* and *rpoD* were selected as the most suitable reference genes among other housekeeping genes of *S. aureus* and *P. aeruginosa*. 10 µL of 2X Syber Green PCR Master Mix (Amplicon, Denmark), 1 µL of each primer (20 pmol), and 2 µL cDNA, and DEPC-treated water was added to each tube to a final volume of 20 µL. The amplification was done based on the following program: 95 °C for 15 min, 40 cycles of 95 °C for 20 s, and 56 °C for 30 s. All the tests were performed in triplicate and in three days.

**Table 4:** List of primers.

Gene	Primer sequence	References
*pvdS*	F: AGATCACTTCGTCGTTCAAGGCA R: GATGTGTTCGAGGGTCGCGTA	^50^
*hasI*	F: TGGATGCCGATGCGCTTG R: CAGCGGGAATCCTCGAGT	^50^
*sigS*	F: ACC TTG AAG GAT ACA AGC AA R:GGC ATT TAC GCT TAA CGG AC	^42^
*fur S. aureus*	F: TTGGAAGAACGATTAA R: TTCTATCCTTTACCTTT	^51^
*fur P. aeruginosa*	F: GGAAGGTATCGACGATCA R:CCCACGCGAAGAAACTG	^52^
*rpoD P. aeruginosa*	F: GGGCTGTCTCGAATACGTTGA R: ACCTGCCGGAGGATATTTCC	^53^
*nucA S. aureus*	F: AGCCAAGCCTTGACGAACTAAAGC R: GCGATTGATGGTGAT ACGGTT	^54^

### Statistical analysis

For all the collected data, a Student t-test was performed by GraphPad Prism 6.0 (Graph Pad Software, USA). The p-values were corrected for multiple testing errors, with a 5% false discovery rate (FDR 5%). The student's t-test was applied to compare the gene expression ratios determined by qRT-PCR between the co-cultures and monocultures. The tests were performed using Holm-Sidak test for multiple average comparisons, considering a p-value of 0.05 or less as significant. All data were presented as mean ± SEM. The gene expression levels were calculated using the 2^−ΔΔCT^ method, and the data were normalized to the reference gene^49^.

### Ethics declarations

The study was conducted under the ethics approval code IR.UMSHA.REC.1399.129.
